# Integrated microbiome and metabolomics analysis reveal the relationship between plant-specialized metabolites and microbial community in *Phellodendron amurense*

**DOI:** 10.3389/fpls.2024.1363063

**Published:** 2024-02-21

**Authors:** Wanran Zhang, Ranran Gao, Lixia Tian, Zhichao Xu

**Affiliations:** ^1^School of Pharmaceutical Sciences, Guizhou University, Guiyang, China; ^2^College of Life Science, Northeast Forestry University, Harbin, China; ^3^Institute of Chinese Materia Medica, China Academy of Chinese Medical Sciences, Beijing, China

**Keywords:** *Phellodendron amurense*, bisbenzylisoquinoline alkaloids, microbial communities, 16S rRNA, ITS

## Abstract

*Phellodendron amurense* is the essential source of bisbenzylisoquinoline alkaloids (BIAs), making it a highly valued raw material in traditional Chinese medicine. The plant’s root secondary metabolism is intricately linked to the microbial communities that surround it. However, the root-associated microbiomes of *P. amurense*, as well as the potential correlation between its bioactive compounds and these microbiomes, remain poorly understood. Here, the metabolic profiles of root, rhizosphere, and bulk soils of *P. amurense* revealed the dramatic differences in the relative content of plant-specialized metabolites. A total of 31, 21, and 0 specialized metabolites in *P. amurense* were identified in the root, rhizosphere soil, and bulk soil, respectively, with higher content of the seven major BIAs observed in the rhizosphere compared with that in the bulk soils. The composition of the bulk and rhizosphere microbiomes was noticeably distinct from that of the endospheric microbiome. The phylum Cyanobacteria accounted for over 60% of the root endosphere communities, and the α-diversity in root was the lowest. Targeted seven BIAs, namely, berberine, palmatine, magnocurarine, phellodendrine, jatrorrhizine, tetrahydropalmatine, and magnoflorine, were significantly positively correlated with Nectriaceae and Sphingobacteriaceae. This study has illuminated the intricate interaction networks between *P. amurense* root-associated microorganisms and their key chemical compounds, providing the theoretical foundation for discovering biological fertilizers and laying the groundwork for cultivating high-quality medicinal plants.

## 1 Introduction

*Phellodendron amurense*, also known as the Amur cork tree, is a tertiary relic plant and belongs to the Rutaceae family (Yu et al., 2013), widely found in Northeastern China, Inner Mongolia, the Russian Far East, southern Sakhalin, Korea, and Japan (Zhang B. Y. et al., 2023). In China, *P. amurense* is mainly distributed in the southeast of the Lesser Khingan, Changbai, and Wanda Mountains and in the northern Yanshan Mountains, the extreme north to Great Khingan Mountains (Yang et al., 2016). The bark of *P. amurense*, a traditional Chinese medicine (TCM), possesses a remarkable medicinal value and has been widely utilized in China, Japan, and Korea (Wang et al., 2015). The deciduous tree is enriched in various chemical compounds such as limonoids, alkaloids, phenolic, polysaccharides, flavonoids, phytosterols, essential oils, and fatty acids (Zhang et al., 2015). The major active ingredients are benzylisoquinoline alkaloids, including berberine, jatrorrhizine, palmatine, magnoflorine, magnocurarine, phellodendrine, and tetrahydropalmatine (Velmurugan et al., 2017; Balážová et al., 2022; Erhan et al., 2023; Feng et al., 2023), which are commonly used as anti-inflammatory, antipyretic, and antibacterial medicines, promoting blood circulation, dysentery, diuretic, hemorrhage, and blood glucose regulating (Kim et al., 2011; Steinmann et al., 2011; Velmurugan et al., 2017; Sun et al., 2019). The yellow, flexible, and tough wood of this medicinal plant boasts an excellent texture and can be used to create furniture and industrial art (Sun et al., 2015). Because of the excessive exploitation for TCM and timber, the population and habitat of wild *P. amurense* have significantly decreased (Ding, 2011). As a result, the Chinese government listed it as a nationally endangered wild plant species (class II) (Fu and Chin, 1992).

The *P. amurense* has been extensively cultivated for use in TCM and extraction of bisbenzylisoquinoline alkaloids (BIAs), but the quality of medicinal materials exhibits noticeable variations across different regions (Zhang et al., 2014; Ma et al., 2015). The quality of Chinese medicinal materials depends on the content and quantity of chemical compositions, whose accumulation is crucial for the pharmacological effects of medicinal plants (Luo et al., 2021; Xiao et al., 2022). A large number of natural products are actually produced by microbe-host interactions, which are directly or indirectly involved in the production of active ingredients (Koberl et al., 2013; Mishra et al., 2022). It also has been confirmed that the quality of traditional herbal medicines is impacted by changes in rhizomicrobiomes and endophytes (Huang et al., 2018; Ling F. et al., 2022). Plant microbiomes could significantly affect the accumulation of key medicinal components, such as alkaloids, terpenoids, and steroids (Pang et al., 2021). *Stenotrophomonas* in the root endosphere of *Polygonum cuspidatum* enhances the emodin content (Zhang Y. H. et al., 2020). Recent research has shown that plant–microbe interactions can improve biomass and tanshinone production in *Salvia miltiorrhiza* (Chen H. M. et al., 2018; Huang et al., 2018). To date, numerous studies have been conducted to investigate the relationship between microbiome communities and bioactive compounds found in medicinal plants (Cui et al., 2019; Zhang et al., 2020; Zhang Y. H. et al., 2020; Li et al., 2021; Cui et al., 2023; Li et al., 2023; Zhang H. et al., 2023). However, the composition characteristics and diversity of rhizosphere and root microbiome communities, as well as their relationship with the major active compounds in *P. amurense*, remain largely unknown.

To address the relationship between the quality of *P. amurense* and its root-associated microbiomes, we studied the metabolite differences among bulk soil, rhizosphere soil, and the root of *P. amurense* and further explore the microbial community composition characteristics, diversity, and biomarker microorganisms of the bulk soil, rhizosphere soil, and endophytic of *P. amurense*. Furthermore, the correlation relationship between the targeted BIAs and the root-associated microbiomes was investigated by weighted gene co-expression network analysis (WGCNA) package in R. Our research aims to offer a practical strategy for enhancing the quality of *P. amurense* by employing an ecological approach to manipulating the root-associated microbiomes.

## 2 Materials and methods

### 2.1 Sample collection

The root and soil of healthy *P. amurense* were collected from Heilongjiang Province, China (45.7662°N, 126.6247°E). Five roots (at a depth of 10–30 cm) from one *P. amurense* were cut off and collected as one sample. In addition, bulk soil (at a depth of 10–20 cm) was collected at a distance of 100–200 cm away from the roots. After removing loose soil from the roots, only 2 mm of rhizosphere soil remained and was collected (Edwards et al., 2015; Hilton et al., 2021). These samples were labeled as R (root of wild *P. amurense*), BS (bulk soil), and RS (rhizosphere soil) with five replicates, respectively. All samples were stored at −80°C. Bulk and rhizosphere soil samples were then sieved by using a 2-mm sieve. The roots of *P. amurense* were carefully washed and sterilized by undergoing the following operations: 70% (v/v) ethanol for 3 min, 2.5% (v/v) sodium hypochlorite (NaClO) for 5 min, and sterile water four times (Wei et al., 2021). Half of each sample was used for the analysis of microbial characteristics, whereas the other half was employed for metabolite detection. Voucher specimens labeled as PAR20221-PAR20225, PABS20221-PABS20225, and PARS20221-PARS20225 were then deposited at the College of Life Sciences, Northeast Forestry University, China.

### 2.2 Determination of targeted BIA metabolites

The collected root samples were dried at 60°C for 1 week, and, then, the dried materials were powered and sieved through a No. 60 mesh. An accurately weighed 0.2 g of sample of the powered materials was extracted ultrasonically with 70% aqueous methanol (2 mL) under 50°C for 1 h, with three replications. The extract was further centrifuged at 12,000 rpm for 10 min, and 1 mL of supernatant was filtered using a 0.22-µm nylon filter. As for the soil sample, 0.2 g of bulk and rhizosphere soil was extracted ultrasonically with 70% aqueous methanol (2 mL) under 50°C for 1 h, with three replications. The extract was centrifuged at 12,000 rpm for 10 min. Supernatant (1 mL) was evaporated (40°C) to dryness in vacuo, and the residue was redissolved in 0.2 mL of 70% methanol. Then, this solution was filtered using a 0.22 nylon filter.

After optimizing the experimental conditions, a method was developed using ultrahigh-performance liquid chromatography (UPLC) coupled with quadrupole time-of-flight mass spectrometry (MS) for the nontarget metabolomic profiling of *P. amurense* and its associated soil. Seven reference standards of phellodendrine, magnoflorine, magnocurarine, jatrorrhizine, tetrahydropalmatine, palmatine, and berberine were dissolved and then diluted with methyl alcohol to prepare a series of standard solutions of different concentrations. To ensure the robustness of the analytical method, the quality control samples were inserted into the sequence every five samples. The samples were injected into a Kinetex C18 column (2.6-μm particle size, 4.6 mm × 150 mm; Phenomenex, USA) maintained at 40°C. The gradient eluent consisted of mobile phase A (0.1% formic acid in water) and mobile phase B (acetonitrile). The gradient elution program was as follows: 0–1 min, 10% B; 1–10 min, 10% to 95% B; 10–12.3 min, 95% B; 12.3–13 min, 95% to 10% B; and 13–15 min 10% B. The flow rate used for separation was 0.5 mL/min, with an injection volume of 1 μL. The MS was equipped with an electrosprayionization (ESI) ion source and operated in the positive ion mode, in the full scan range from mass-to-charge ratio (m/z) of 100 to 2,000 with fragmentation of the five most intensive signals. The following MS conditions were used: ion spray voltage, 5,500 V; curtain gas, 25 psi; ion source gas 1, 40 psi; ion source gas 2, 60 psi; and temperature, 550°C.

The mass spectrum data were analyzed by Analyst TF1.8 Software (https://sciex.com/products/software/analyst-software/). Using the metabolome software LibraryView (AB Sciex, Shanghai, China), the samples’ metabolites were analyzed qualitatively and quantitatively. The chemical structure of the compounds was characterized on the basis of the calculated accurate masses of the molecular ions, protonated molecules, fragment ions, and retention behavior. The observed constituent ion peaks [M]^+^/[M+H]^+^/[M+NH4]^+^ provide reliable information that can confirm the molecular weights and structures of the compounds. The relative contents (after taking log values) of compounds in different samples were displayed by R package pheatmap (v1.0.12).

### 2.3 DNA extraction and sequencing

Metagenomic DNA from root, rhizosphere soil, and bulk soil samples was extracted using the HiPure Soil DNA Kits (Magen, Guangzhou, China) according to the manufacturer’s recommendations. The DNA concentration and purity were accurately measured using the NanoDrop 2000 spectrophotometer prior to performing agarose (1% w/v) gel electrophoresis. The purified DNA was used to amplify either the fungal Internal Transcribed Spacer 2 (ITS2) region ITS3_KYO2 (5′-GATGAAGAACGYAGYRAA-3′) and ITS4 (5′-TCCTCCGCTTATTGATATGC-3′) (Guo et al., 2017) or the V3-V4 region of the bacterial 16S ribosomal RNA (rRNA) gene 341F (5′-CCT ACG GGNGGC WGC AG-3′) and 806R (5′-GGAC TAC HVGGG TAT CTA AT-3′) (Toju et al., 2012). The PCR-negative controls consisted of ddH_2_O instead of DNA template. All samples were subjected to the following PCR conditions: initial denaturation at 95°C for 2 min, followed by 27 cycles of denaturation at 98°C for 10 s, annealing at 62°C for 30 s, extension at 68°C for 30 s, and a final extension at 68°C for 10 min. The paired-end approach was utilized for sequencing by using the Illumina Novaseq 6000 sequencing platform.

### 2.4 Characteristic analysis of the microbial communities

To obtain high-quality clean reads, raw reads were further filtered using FASTP (v0.18.0) (Chen S. F. et al., 2018). Paired-end clean reads were merged into raw tags using FLASH (v1.2.11) (Magoc and Salzberg, 2011) with a minimum overlap of 10 bp and mismatch error rates of 2%. The clean tags were clustered into operational taxonomic units (OTUs) of ≥ 97% similarity using UPARSE (v9.2.64) (Edgar, 2013), a high-accuracy and high-throughput OTU clustering pipeline. In each cluster, the tag sequence with the highest abundance was selected as the representative sequence.

The stacked bar plot of the community composition was visualized in the ggplot2 package (v2.2.1) (Wicham, 2011), and circular layout representations of species abundance at the genus level were created using circos (v0.69-3) (Krzywinski et al., 2009). To evaluate the diversity of species and community of the bacterial and fungal microorganisms in roots, rhizosphere soil, and bulk soil of *P. amurense*, alpha-diversity based on the diversity index and beta-diversity based on the weighted UniFrac distance matrix were conducted, respectively. Chao1, Abundance-based Coverage Estimator (ACE), Shannon, and Simpson index were calculated using QIIME (v1.9.1) (Caporaso et al., 2010), and PCoA (principal coordinate analysis) (Dixon, 2003) was generated using the Vegan package (v2.5.3) and ggplot2 package (v2.2.1) of R project. The biomarker features in bacterial and fungal microorganism communities of *P. amurense* were evaluated through Linear discriminant analysis Effect Size (LEfSe) (Segata et al., 2011).

### 2.5 Correlation analyses of the main active compounds and microbial communities

WGCNA was performed to evaluate the correlation between the secondary metabolites in roots and their associated microbial communities. After obtaining the absolute contents of seven key BIAs in the bulk soil, rhizosphere soil, and roots of *P. amurense*, combined with the abundance of bacteria and fungi OTU in the bulk soil, rhizosphere soil, and roots of *P. amurense*, co-expression network modules were generated by WGCNA package in R. Eigengene value was calculated for each module to search the family-level microorganisms associated with BIA metabolism in *P. amurense*. The co-expression modules were obtained using automatic network construction function (blockwiseModules) with default parameters; apart from the soft threshold power of 18, networkType was unsigned, TOMtype was unsigned, mergeCutHeight was 0.1, and minModuleSize was 15. In addition, the Pearson correlation coefficient was also calculated. Microorganisms and metabolites with a correlation greater than 0.9 and *P* < 0.01 were selected, and the correlation network was drawn using Cytoscape (v3.9.1, https://cytoscape.org/).

## 3 Results

### 3.1 Distribution of targeted metabolites in the roots and rhizosphere soils of *P. amurense*

**Figure 1:**
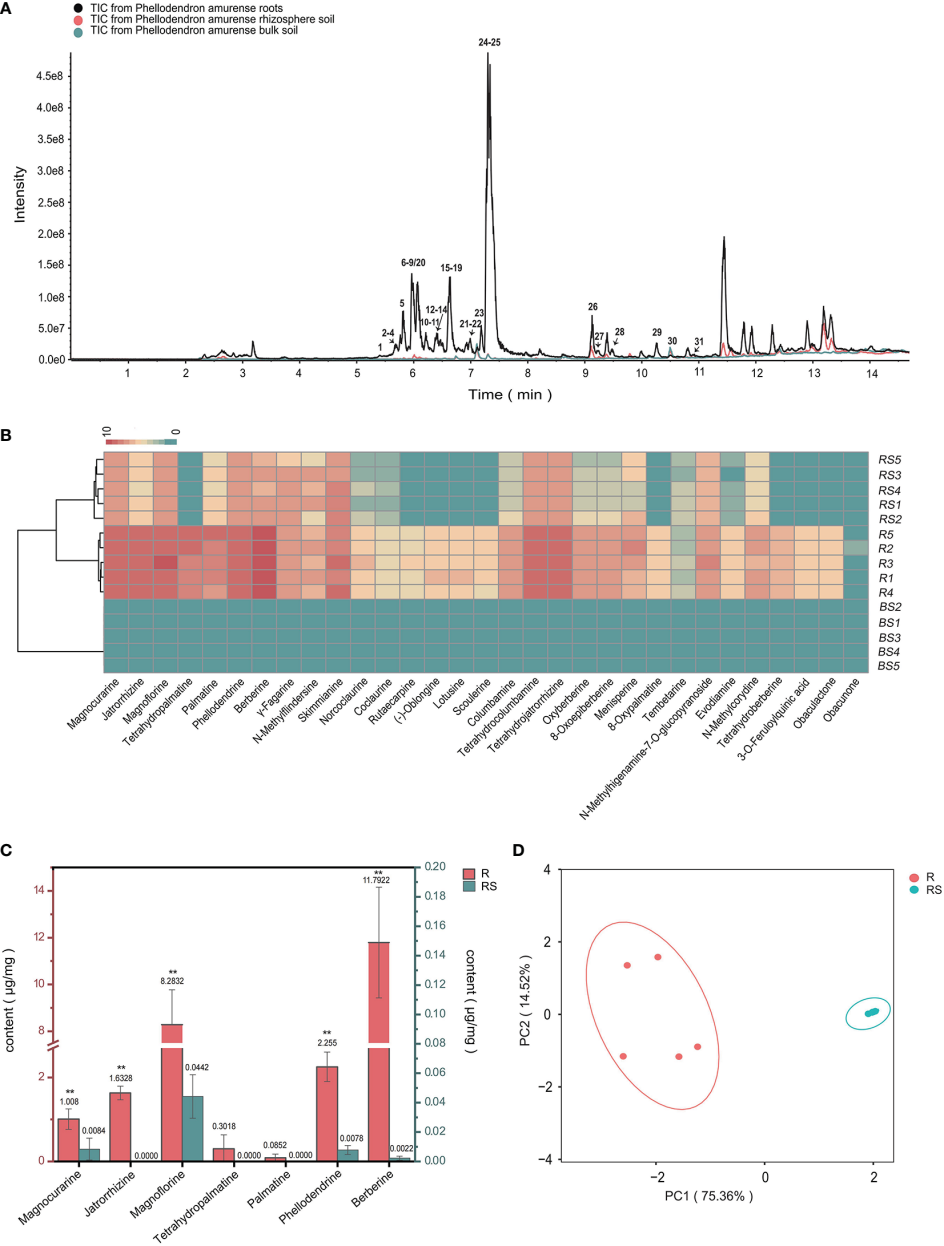
Metabolic analyses of *Phellodendron amurense* roots (R), rhizosphere soil (RS), and bulk soil (BS). **(A)** ESI-MS total ion chromatogram profiles of the *P. amurense* extracts. **(B)** UPLC-ESI-MS/MS identification of the compounds contained in 15 samples of *P. amurense* is shown in heatmap. **(C)** The histogram of seven main alkaloids in *P. amurense* roots and rhizosphere soil. The red scale (left) is used to measure the absolute content of active ingredients in roots, and, similarly, the blue scale (right) is used to measure the absolute content of bioactive compounds in rhizosphere soil. *p < 0.05 and **p < 0.01. **(D)** Principal component analysis (PCA) of roots and rhizosphere soil.

To uncover the distribution characteristics of main active compounds of bulk, rhizosphere soil, and root of *P. amurense*, the targeted metabolomics was employed, which focuses on the analysis of specific categories of metabolites with more selectivity and sensitivity (Roberts et al., 2012). Obvious differences in the quantity and relative content of specialized metabolites were observed at the different compartments of *P. amurense*. A total of 31 metabolites were detected and identified (**Table 1**, **Figure 1A**). These metabolites include 28 alkaloids, two limonoids, and one quinic acid. Among the 28 alkaloids, 23 are BIAs, two are indole-type, two are quinoline-type, and one is another type of alkaloid. The 23 BIAs can be classified into three groups: three aporphine alkaloids, seven benzylisoquinoline alkaloids, and 13 protoberberine alkaloids (**Supplementary Table S1**, **Supplementary Information**). The number of BIAs is the greatest among the 28 alkaloids, accounting for 82% of all the BIA metabolites. In addition, 31 specialized compounds were characterized in the root of *P. amurense*, whereas 21 were shared in the rhizosphere soil. No specialized metabolites of *P. amurense* were detected in the bulk soil (**Figure 1B**). There was a significantly higher content of the 21 commonly specialized compounds in the roots compared with that in the rhizosphere soil.

**Table 1:** UPLC-ESI-MS/MS identification of the compounds contained in roots and rhizosphere soil of P. amurense in a positive mode.

No.	t_R_^b^ (min)	Identification	Molecular formula	Parent ion^c^ (*m/z*)	Fragmentation profile (*m/z*)
1	5.31	Norcoclaurine *	C_16_H_17_NO_3_	272.1281 [M+H]^+^	107.0495, 161.0595, 123.0439, 143.0490
2	5.67	*N*-Methylhigenamine-7-O-glucopyranoside		448.1901 [M+H]+	286.1400, 255.0968
3	5.71	(−)-Oblongine	C_19_H_24_NO_3_	315.1829 [M+H]^+^	107.0464, 270.1165, 176.0743
4	5.71	Lotusine	C_19_H_24_NO_3_	315.1829 [M+H]^+^	107.0464, 270.1165, 176.0743
5	5.81	Phellodendrine *	C_20_H_24_NO_4_	342.1692 [M]^+^	177.0815, 192.1117, 148.0730, 190.0826
6	5.91	Tembetarine *	C20H26NO4	344.1814 [M]+	267.0959, 192.0980, 177.0856
7	5.97	Magnoflorine *	C_20_H_24_NO_4_	342.1687 [M]^+^	265.0832, 297.1063, 237.0860, 282.0836
8	5.97	Tetrahydrocolumbamine *	C_20_H_23_NO_4_	342.1654 [M+H]^+^	297.1063, 265.0832, 282.0836, 237.0860
9	5.97	Tetrahydrojatrorrhizine *	C_20_H_23_NO_4_	342.1654 [M+H]^+^	297.1063, 265.0832, 282.0836, 237.0860
10	6.22	Magnocurarine *	C_19_H_24_NO_3_	314.1551 [M]^+^	107.0471, 237.0860, 254.0872
11	6.24	8-Oxopalmatine *	C_21_H_21_NO_5_	368.1493 [M+H]^+^	338.0950, 353.1182, 310.1009
12	6.37	3-O-Feruloylquinic acid	C_17_H_20_O_9_	386.1400 [M+NH4]^+^	177.0506, 117.0304
13	6.40	(+) N-Methylcorydine *	C_21_H_26_NO_4_	356.1785 [M]^+^	311.1203, 279.0955, 251.1007
14	6.40	Menisperine *	C_21_H_26_NO_4_	356.1935 [M]^+^	264.0727, 279.0955, 248.0781, 296.0978
15	6.61	Evodiamine *	C_19_H_17_N_3_O	304.1474 [M+H]^+^	144.0768, 128.0578
16	6.62	Coclaurine *	C_17_H_19_NO_3_	286.1338 [M+H]^+^	256.0913, 271.1129, 254.1120
17	6.65	Tetrahydropalmatine	C_21_H_25_NO_4_	356.1863 [M+H]^+^	192.1119, 356.1774, 190.0821
18	6.72	8-Oxoberberine *	C_20_H_17_NO_5_	352.1104 [M+H]^+^	337.0877, 308.0852, 322.0641, 294.0696
19	6.72	8-Oxoepiberberine *	C_20_H_17_NO_5_	352.1104 [M+H]^+^	337.0877, 308.0852, 322.0641, 294.0696
20	5.97	Scoulerine	C_19_H_21_NO_4_	328.2 [M+H]^+^	177.0717, 121.0618, 144.0524
21	6.97	Jatrorrhizine *	C_20_H_20_NO_4_	338.1388 [M]^+^	323.1077, 322.1019, 294.1061, 265.0685
22	6.97	Columbamine *	C_20_H_20_NO_4_	338.1388 [M]^+^	323.1077, 322.1019, 294.1061, 265.0685
23	7.18	Tetrahydroberberine	C_20_H_21_NO_4_	340.1467 [M+H]^+^	176.0666,149.0563, 340.1469
24	7.26	Palmatine *	C_21_H_22_NO_4_	352.1553 [M]^+^	336.1167, 308.1211, 294.1054, 322.1002
25	7.30	Berberine *	C_20_H_18_NO_4_	336.1224 [M]^+^	320.1004, 278.0842, 292.1038, 306.0777
26	9.13	Skimmianine *	C_14_H_13_NO_4_	260.0918 [M+H]^+^	227.0523, 199.0604, 216.0600, 184.0357
27	9.26	γ-Fagarine *	C_13_H_11_NO_3_	230.0812 [M+H]^+^	215.0523, 200.0295
28	9.46	Obaculactone	C_26_H_30_O_8_	488.2155 [M+NH4]^+^	425.1851, 161.0555, 367.1862
29	10.27	Obacunone	C_26_H_30_O_7_	455.1970 [M+H]^+^	427.1951, 409.1892
30	10.60	Rutaecarpine	C_18_H_13_N_3_O	288.1132 [M+H]^+^	273.0808, 169.0711, 243.0841, 144.0750
31	10.91	*N*-Methylflindersine *	C_15_H_15_NO_2_	242.1176 [M+H]^+^	212.0652, 188.0654, 226.0806

* indicates that the compound was detected on rhizosphere soil samples. All the chemical compounds can be identified in root.

To determine the major chemical compounds present in the rhizosphere soil and roots of *P. amurense*, we used UPLC-MS/MS to analyze the content of seven BIAs (phellodendrine, magnoflorine, magnocurarine, jatrorrhizine, tetrahydropalmatine, palmatine, and berberine), which have been shown to have well pharmacological activity. The highest contents of magnoflorine and berberine were found in the roots, whereas magnoflorine, magnocurarine, and phellodendrine were the main compounds in the rhizosphere soil (**Figure 1C**). The content of the seven BIAs in roots was significantly higher than that in rhizosphere soil, and these BIAs in roots and rhizosphere soil were, respectively, clustered in two groups, suggesting well sample repetition (**Figure 1D**).

### 3.2 Composition of *P. amurense* root-associated microbial communities

**Figure 2:**
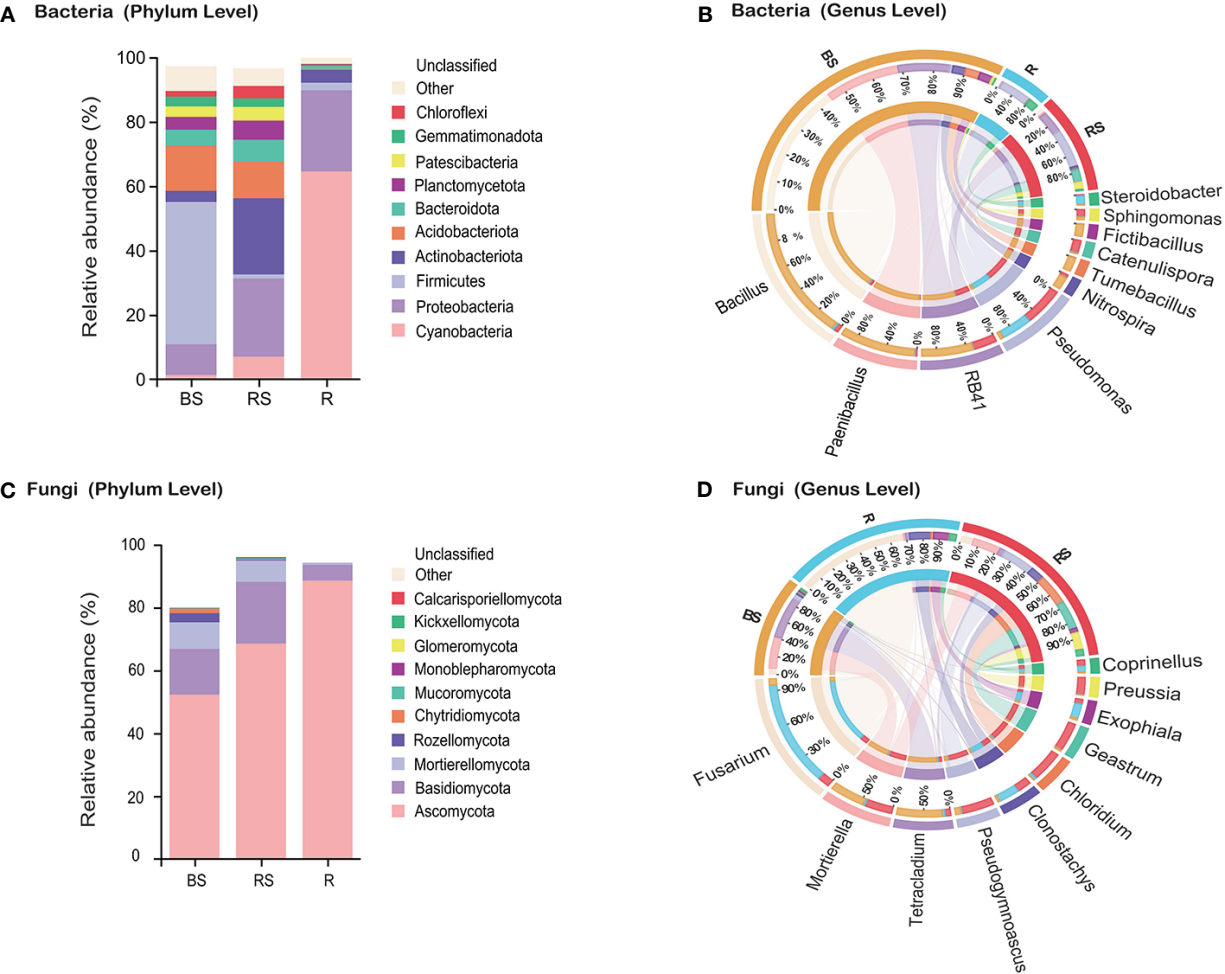
Microbiota community composition of bacteria and fungi in *Phellodendron amurense* roots (R), rhizosphere soil (RS), and bulk soil (BS). (**A, C**) The relative abundance of bacteria and fungi at the phylum levels among different samples. (**B, D**) Circos plot showing the distribution proportion of core microbiota at the genus levels among different samples.

To understand the differences in microbial composition among the bulk, rhizosphere soil, and root, statistical analyses were performed on different taxonomic levels. By using a cutoff of >97% sequence identity, high-quality reads were clustered into bacterial 15,950 OTUs, 16,471 OTUs, and 4,452 OTUs of bulk, rhizosphere soil, and root, respectively. Regarding fungi, high-quality reads were clustered into 3,359 OTUs, 3,526 OTUs, and 1,503 OTUs of bulk, rhizosphere soil, and root, respectively. Rarefaction curve analysis revealed that all samples were nearly parallel to the X-axis, suggesting that the obtained sequences adequately depict the overall bacterial and fungal diversity (**Supplementary Figure S1**, **Supplementary Information**). A taxonomic histogram showed that the composition of *P. amurense* root-associated microbial communities varied along with the compartments. At the bacterial phylum level, the microbial composition analysis revealed that Firmicutes, Acidobacteriota, and Proteobacteria exhibited the highest abundance in bulk soil, whereas Actinobacteriota, Proteobacteria, and Acidobacteriota were enriched in rhizosphere soil (**Figure 2A**). Notably, the root microbiota was characterized by a prevalence of Cyanobacteria, Proteobacteria, and Actinobacteriota. A marked increase in the proportion of the dominant Cyanobacteria species was observed in roots. At the genus level, the predominant bacterial genera in bulk soil were *Bacillus* (21.68%), *Paenibacillus* (11.52%), and *RB41* (7.90%). In rhizosphere soil, *Pseudomonas* (6.23%), *RB41* (3.63%), and *Catenulispora* (2.10%) exhibited the highest proportions. Similarly, *Pseudomonas* (4.76%), *Steroidobacter* (1.46%), and *Bacillus* (0.34%) were identified as the top three bacterial genera in the root (**Figure 2B**).

Regarding fungi, the phyla Ascomycota, Basidiomycota, and Mortierellomycota were identified as the most abundant, aligning with a globally observed soil pattern (**Figure 2C**). Analysis of the relative abundance of fungal genera indicated higher levels of *Fusarium* (BS, 1.71%; RS, 3.04%; and R, 27.71%), *Clonostachys* (BS, 0.50%; RS, 3.51%; and R, 5.15%), and *Exophiala* (BS, 0.51%; RS, 1.26%; and R, 3.89%) in the roots compared with that in the bulk soil and rhizosphere soil (**Figure 2D**). Conversely, the relative abundance of fungal genera *Mortierella* (BS, 8.48%; RS, 6.79%; and R, 0.52%), *Tetracladium* (BS, 11.04%; RS, 1.37%; and R, 0.68%), and *Pseudogymnoascus* (BS, 1.33%; RS, 7.90%; and R, 0.25%) demonstrated higher levels in the rhizosphere soil and bulk soil compared with that in the root.

### 3.3 Diversity of *P. amurense* root-associated microbial communities

**Figure 3:**
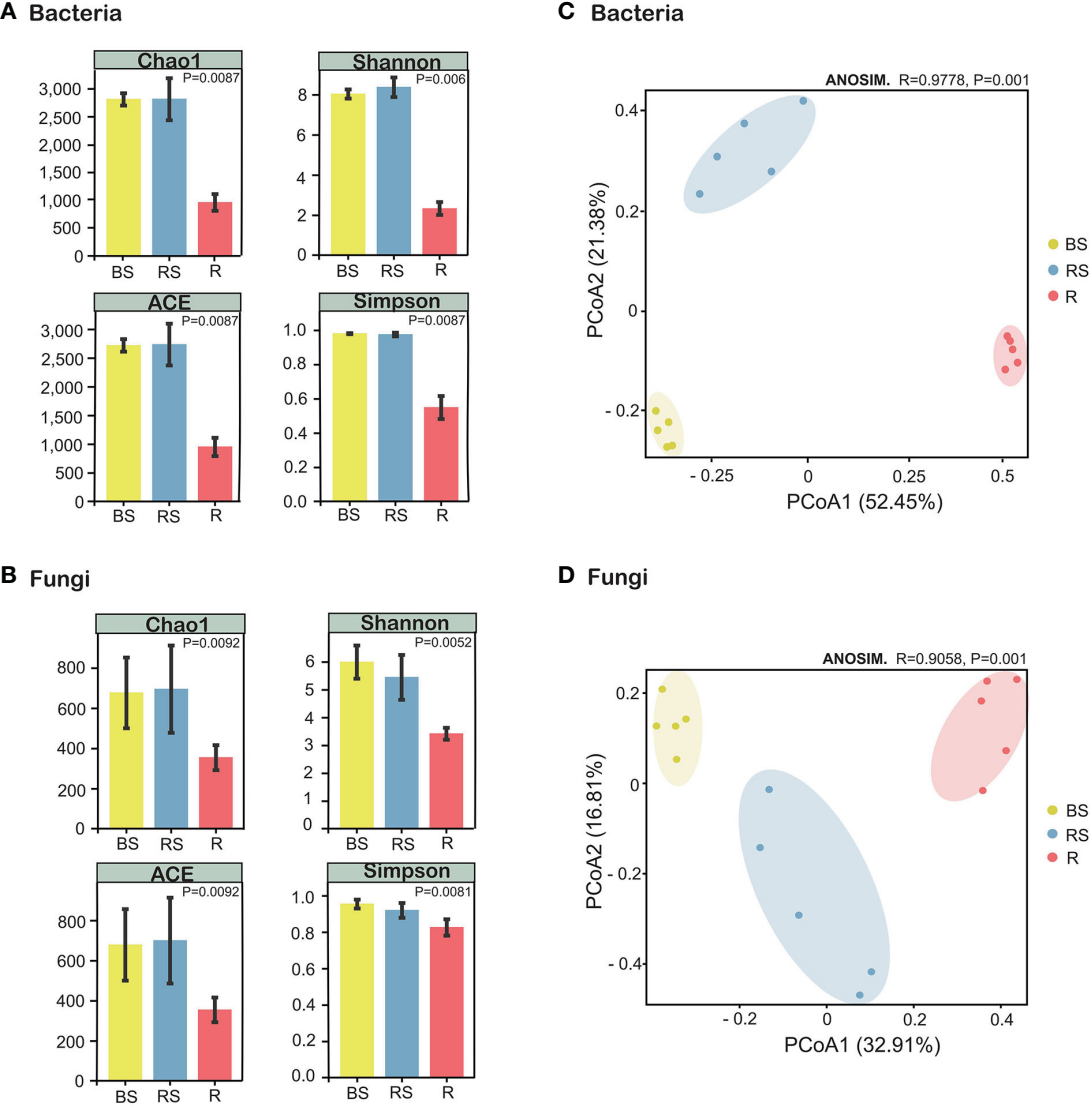
Alpha- and beta-diversity indices of the microorganisms in *Phellodendron amurense* roots (R), rhizosphere soil (RS), and bulk soil (BS). **(A)** Chao1 index, ACE index, Shannon index, and Simpson index of the bacterial communities. **(B)** Chao1 index, ACE index, Shannon index, and Simpson index of the fungal communities. **(C)** Principal coordinate analysis (PCoA) of the bacterial communities. **(D)** PCoA plot of the fungal communities.

The α-diversity index serves as a quantitative metric for assessing species richness within microbial communities, with higher diversity being considered advantageous for overall soil health. Analysis of α-diversity revealed significant variations among sampling compartments (bulk, rhizosphere soil, and root), demonstrating higher diversity in the bulk soil and rhizosphere soil compared with that in root. The Simpson, Chao1, ACE, and Shannon indices for both bacteria and fungi exhibited a sequential decrease in the order of bulk, rhizosphere soil, and root, with the α-diversity in root being the lowest (**Figures 3A, B**). Kruskal–Wallis analysis further confirmed α-diversity significant differences (*p* < 0.01) among bulk, rhizosphere soil, and root for both bacteria and fungi (**Supplementary Figure S2**, **Supplementary Information**). For β-diversity, PCoA using Bray–Curtis distance assessed the compositional relatedness of bacterial and fungal communities. Results indicated distinct separation of microbiome communities across different sampling compartments. In the case of bacteria, PCoA1 and PCoA2 accounted for 52.45% and 21.38% of the total variance, respectively, with the cumulative variance of PCoA contributing to 73.83% (**Figure 3C**). Similarly, for fungi, PCoA1 and PCoA2 explained 32.91% and 16.81% of the total variance, respectively (**Figure 3D**). The PCoA analysis underscored pronounced differences in the community composition of bacteria and fungi among bulk, rhizosphere soil, and root. Collectively, these findings emphasize the distinct microbial community compositions across the sampled compartments, underscoring the ecological heterogeneity within the studied soil system.

### 3.4 Potential biomarkers of *P. amurense* root-associated microbial communities

**Figure 4:**
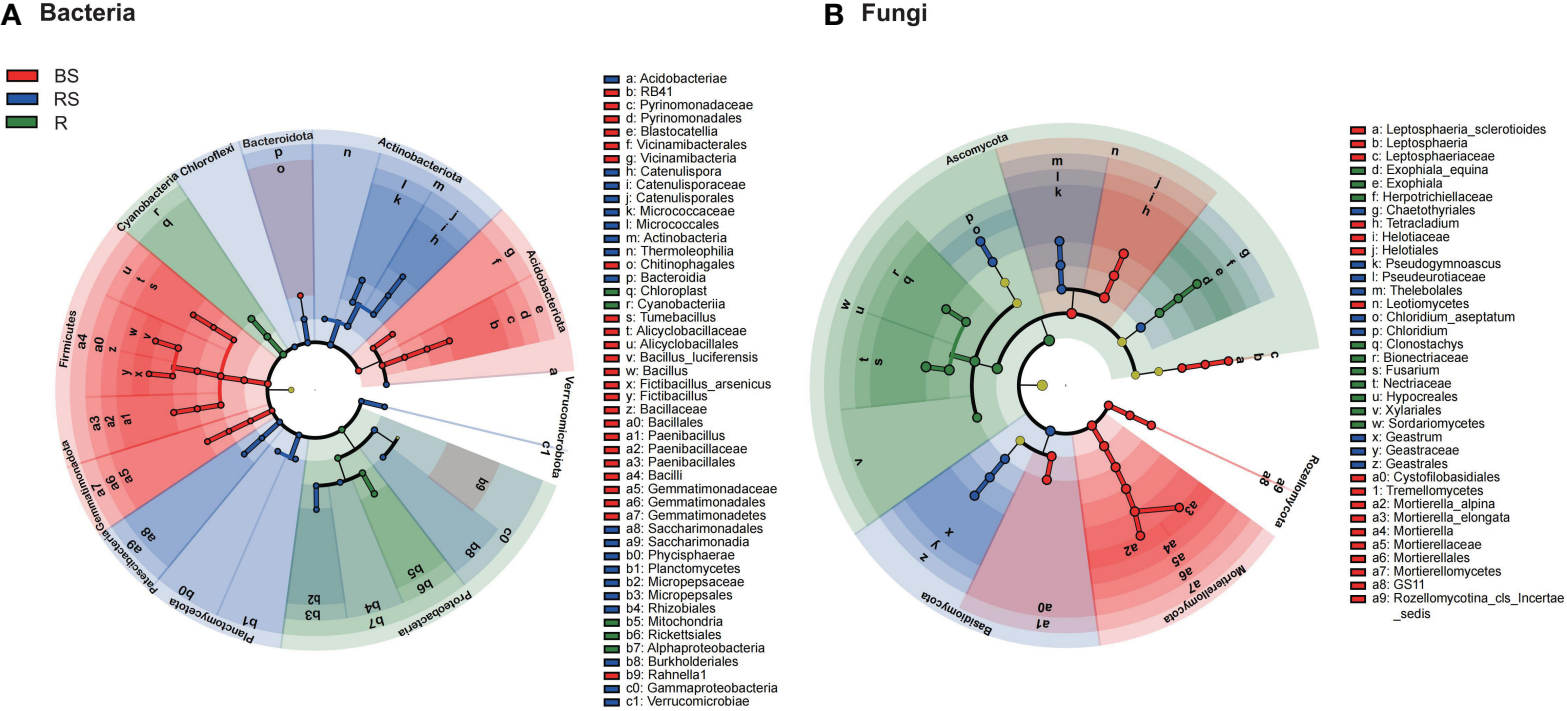
The cladograms of taxa with significant differences in *Phellodendron amurense* roots (R), rhizosphere soil (RS), and bulk soil (BS). **(A, B)** Circular tree plot generated from LEfSe analysis of bacteria and fungi with a linear discriminant analysis (LDA) score higher than 4.0 and p-values less than 0.05.

The LEfSe methods were employed to discern features exhibiting significant differential abundance among bulk, rhizosphere soil, and root, subsequently determining biomarker microbiota at the family level with a stringent criterion of linear discriminant analysis (LDA) score > 4 (**Supplementary Figure S3**). For bacteria (**Figure 4A**), the LDA scores for Bacillaceae and Paenibacillaceae were most elevated in bulk soil, whereas those for Micrococcaceae and Micropepsaceae peaked in rhizosphere soil. Notably, no bacterial biomarkers were annotated at the family level in roots with an LDA score exceeding 4. In the fungi (**Figure 4B**), the family-level LDA scores revealed distinct biomarkers. Helotiaceae and Mortierellaceae exhibited markedly higher scores in bulk soil, whereas Geastraceae and Pseudeurotiaceae dominated in rhizosphere soil. On the other hand, roots featured a higher abundance of Nectriaceae and Bionectriaceae. Consequently, we identified 17 biomarker microbial families, namely, Bacillaceae, Paenibacillaceae, Pyrinomonadaceae, Gemmatimonadaceae, Alicyclobacillaceae, Alicyclobacillaceae, Micrococcaceae, Micropepsaceae, Catenulisporaceae, Helotiaceae, Mortierellaceae, Leptosphaeriaceae, Geastraceae, Pseudeurotiaceae, Nectriaceae, Bionectriaceae, and Herpotrichiellaceae. These biomarkers contribute to the distinctive microbial signatures characterizing bulk, rhizosphere soil, and root in the studied soil ecosystem.

### 3.5 Correlation analyses of the main active compounds and microbial communities

**Figure 5:**
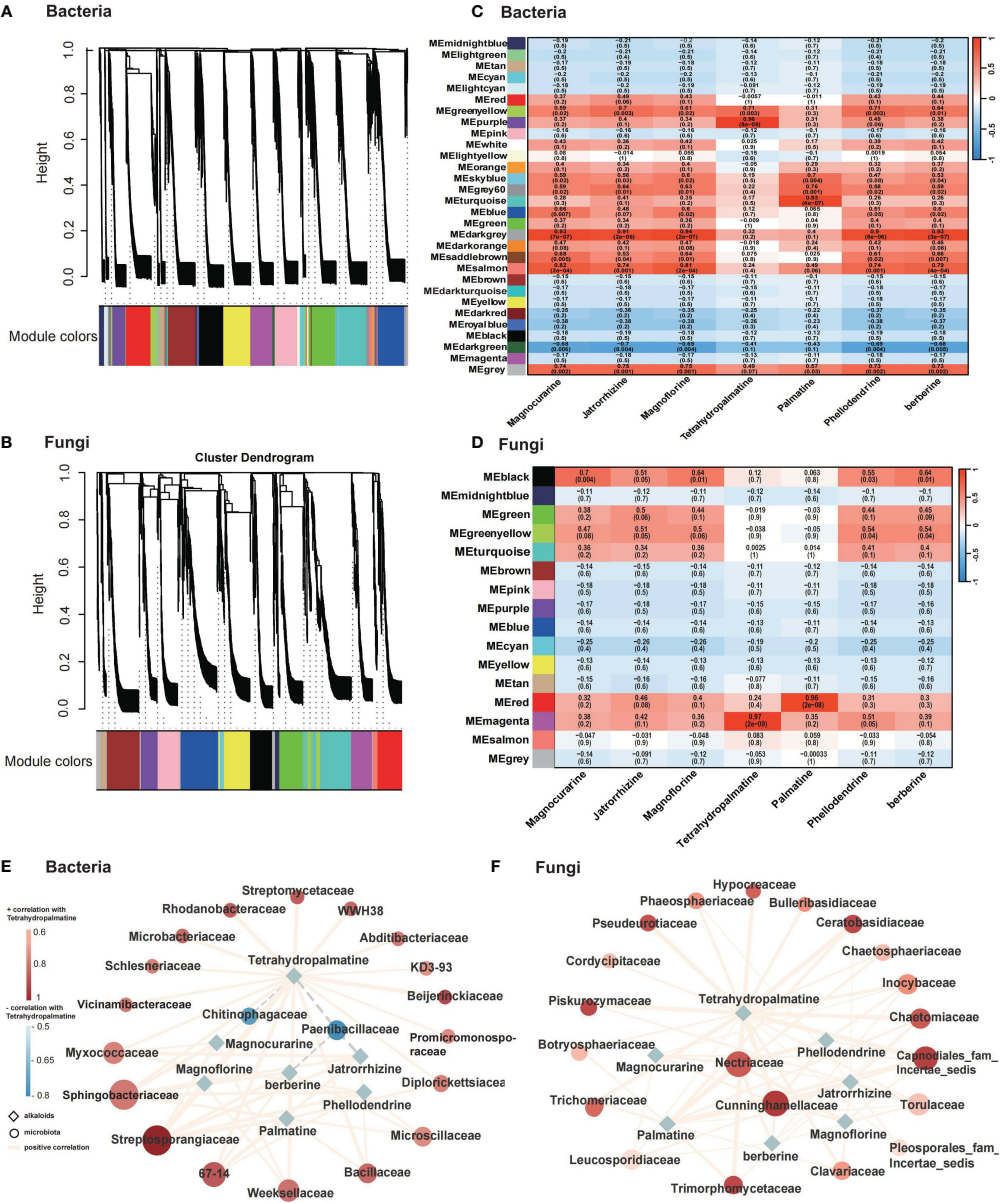
Microbial and metabolic correlation analysis across roots (R), rhizosphere soil (RS), and bulk soil (BS) samples of *Phellodendron amurense*. **(A, B)** Dendrogram showing co-expression modules (clusters) identified by weighted correlation network analysis (WGCNA) across plant root compartments and bulk soil. **(C, D)** Heatmap showing module–alkaloid correlations of bacteria and fungi in *P. amurense*. Each row corresponds to a module indicated by different colors, represents a cluster of OTUs with similarity over 90%. Each column corresponds to an alkaloid. **(E, F)** Metabolism of main alkaloids and top 20 putative bacteria and fungi microorganisms’ regulatory network (P < 0.01). The solid line (orange) and dotted line (gray) indicate positive and negative correlations, respectively. The shape of the node denotes the components used in this study (ellipse, microbiome; diamond, alkaloids), and the color indicates the degree of correlation with tetrahydropalmatine.

To unravel the correlation between microbiome communities and the principal bioactive chemical compounds in *P. amurense*, a total of 4,717 bacterial OTUs and 1,344 fungal OTUs from roots, rhizosphere soil, and bulk soil, alongside data on the seven main alkaloids, were utilized to construct a WGCNA network. This analysis identified 30 and 16 co-expression modules for bacteria and fungi, respectively, based on their similar expression patterns (**Figures 5A, B**). In bacteria, MEpurple is significantly related to Tetrahydropalmatine. Additonally, the module MEdarkgrey is significantly correlated with Magnoflorine; And the module MEturquoise is significantly related to Palmatine (**Figure 5C**). In fungi, MEmagenta is significantly related to Tetrahydropalmatine and the module MEred is significantly related to the Palmatine (**Figure 5D**). Subsequently, correlation analysis between key microorganisms within these modules and the seven BIAs of *P. amurense* was conducted, and the networks were visualized using Cytoscape. Regarding bacteria, all seven benzylisoquinoline alkaloids displayed significantly positive correlations (*p* < 0.01) with Streptosporangiaceae and Sphingobacteriaceae (**Figure 5E**). However, Chitinophagaceae showed a negative correlation with tetrahydropalmatine, whereas Paenibacillaceae exhibited negative correlations with tetrahydropalmatine, berberine, and jatrorrhizine. In fungi, berberine, palmatine, jatrorrhizine, phellodendrine, tetrahydropalmatine, magnocurarine, and magnoflorine exhibited significantly positive correlations (*p* < 0.01) with Nectriaceae and Cunninghamellaceae (**Figure 5F**). These findings provide insights into the intricate interplay between microbial communities and the biosynthesis of bioactive compounds in *P. amurense*.

## 4 Discussion

*P. amurense* may exudate specialized BIA metabolites into rhizosphere soil. The rhizosphere refers to the narrow zone of soil that is influenced by root secretions. The present study revealed that the relative content of root alkaloids was higher than that in rhizosphere soil, with bulk soil being not detected. In a study of pepper greenhouse cultivation, it was observed that metabolites were differ between rhizosphere soil and bulk soil, with the majority of metabolites being more abundance in rhizosphere soil compared with that in bulk soil (Song et al., 2020). We observed the similar results in *P. amurense*, and these differences were primarily observed in plant alkaloid metabolites, which were attributed to the biological properties of *P. amurense*. *P. amurense* mainly accumulates BIAs, especially in protoberberine-type alkaloids, including berberine, palmatine, jatrorrhizine, phellodendrine, and tetrahydropalmatine (Wang et al., 2015). This finding supported that plant root-released metabolites play an essential role in the metabolites profiles in rhizosphere soil (Zhang H. L. et al., 2020).

Various studies have demonstrated that medicinal plants can impact their microbial composition by releasing different types of metabolites (Hu et al., 2018; Nakayasu et al., 2021; Sugiyama, 2021). The root-associated bacterial and fungal communities were found to be distinctly different in the three soil-root compartments. This could be due to the fact that the root exudates of *P. amurense* have a greater impact on rhizosphere bacteria than on bulk soil bacteria. The higher input of rhizodeposits into the rhizosphere may lead to significant shift in the bacterial community. The fungi communities associated with the roots in *P. amurense* were similar to previous studies, mainly including Ascomycota, Basidiomycota, and Mortierellomycota, regardless of plant species (Muller et al., 2016; Trivedi et al., 2020; Liu et al., 2023). In addition, the α-diversity of soil bacteria and fungi was higher in the bulk soil than that in the rhizosphere, with the lowest diversity found in the roots. This result is consistent with other studies that explain how plants select their ecological niche (Trivedi et al., 2020; Xiong et al., 2021; Liu et al., 2022). This may be due to the fact that bulk soil can be compared to a “microbial seed bank,” serving as a reservoir from which rhizosphere microorganisms are recruited. As a result, the diversity of rhizosphere microorganisms is reduced (Ling N. et al., 2022).

In the present study, the phylum Cyanobacteria constituted more than 60% of the root endosphere communities. It is noteworthy that some Cyanobacteria have the ability to fix nitrogen through photosynthesis, and inner diazotrophic bacteria have been well documented in rice, maize, sugarcane, and some gramineous energy plants (Roesch et al., 2007; Thaweenut et al., 2010; Zhang et al., 2019; Purwani et al., 2021). Nitrogen-fixing microorganism is a kind of microorganism that can convert nitrogen into ammonia. Nitrogen-fixing microorganism plays an important role in nature and can convert nitrogen in the atmosphere into ammonia that can be absorbed and utilized by plants, which plays an important role in maintaining ecological balance and promoting plant growth. Alkaloids are a class of nitrogen-containing organic compounds existing in organisms, which usually have complex structures and diverse biological activities. Alkaloid components are formed by the amino acid pathway, and amino acids decarboxylated into amines, which are converted into alkaloids through a series of chemical reactions (methylation, oxidation, reduction, rearrangement, etc.). Therefore, it is speculated by our study that nitrogen-fixing microorganisms may provide nitrogen source for alkaloid biosynthesis by fixing more nitrogen, resulting in a higher alkaloid content in *phellodendron amurense*. However, this hypothesis requires further experimental verification.

In this study, significant relationships were observed between specific microbes and the contents of bioactive compounds. Nectriaceae was significantly positively correlated with seven BIAs, including berberine, palmatine, jatrorrhizine, phellodendrine, tetrahydropalmatine, magnocurarine, and magnoflorine. Nectriaceae was also the biomarker of root compared with that of bulk and rhizosphere soil in LEfSe analysis. It has been reported that members of Nectriaceae family are commonly found in various environments, where they play significant socio-economic roles in agriculture, industry, and medicine. The majority of these species are soil-borne saprobes or weak to virulent, facultative, or obligate plant pathogens, whereas some are facultatively fungicolous or insecticolous (Lombard et al., 2015).

Furthermore, seven BIAs showed a significant positive correlation with Sphingobacteriaceae. These bacteria are commonly found in nature, particularly in soils, oceans, and freshwater, due to their ability to utilize a variety of organic compounds and to thrive and survive under low-nutrient conditions. Sphingobacteriaceae, a member of Sphingobacteriales, plays a significant role as a plant growth-promoting rhizobacteria that can enhance the growth of rice and tomato (Asaf et al., 2018). In addition, it serves as an eco-friendly biological resource for decontaminating polluted areas while promoting the growth of plants facing environmental disturbances (Khan et al., 2014; Kim et al., 2019). In the next step, inoculation experiments will be conducted to clarify the mechanism by which the specific microbes stimulate the production of plant specialized metabolites in *P. amurense.*

## 5 Conclusions

Here, we investigated the rhizosphere soil and root metabolites of the medicinal plant *P. amurense*, as well as their microbiome characteristics, and decoded the relationship between the microbial communities and the seven root-derived specialized metabolites. These metabolites varied significantly among the *P. amurense* compartments. The composition of the rhizosphere microbiome significantly differed from that of the endospheric microbiome. Seven benzylisoquinoline alkaloids (berberine, palmatine, jatrorrhizine, phellodendrine, tetrahydropalmatine, magnocurarine, and magnoflorine) were significantly positive correlated with Nectriaceae and Cunninghamellaceae. In summary, this study has revealed a complex interplay between root-associated microorganisms and plant secondary metabolites, offering a potential strategy for enhancing the industrial and pharmacological value of *P. amurense*.

## Data availability statement

The datasets presented in this study can be found in online repositories. The names of the repository/repositories and accession number(s) can be found below: BioProject, PRJNA1066709 and PRJNA1066243.

## Author contributions

LT: Conceptualization, Funding acquisition, Supervision, Writing – review & editing. ZX: Conceptualization, Funding acquisition, Resources, Supervision, Writing – review & editing. RG: Data curation, Funding acquisition, Methodology, Writing – review & editing. WZ: Formal Analysis, Investigation, Validation, Visualization, Writing – original draft.
