# Supplementary material for: Integrated microbiome and metabolomics analysis reveal the relationship between plant-specialized metabolites and microbial community in Phellodendron amurense
Source: Front Plant Sci. 2024 Feb 21;15:1363063. doi: 10.3389/fpls.2024.1363063 (PMC10915045; doi:10.3389/fpls.2024.1363063)
Supplement: Supplementary file 1 [file DataSheet_1.pdf]

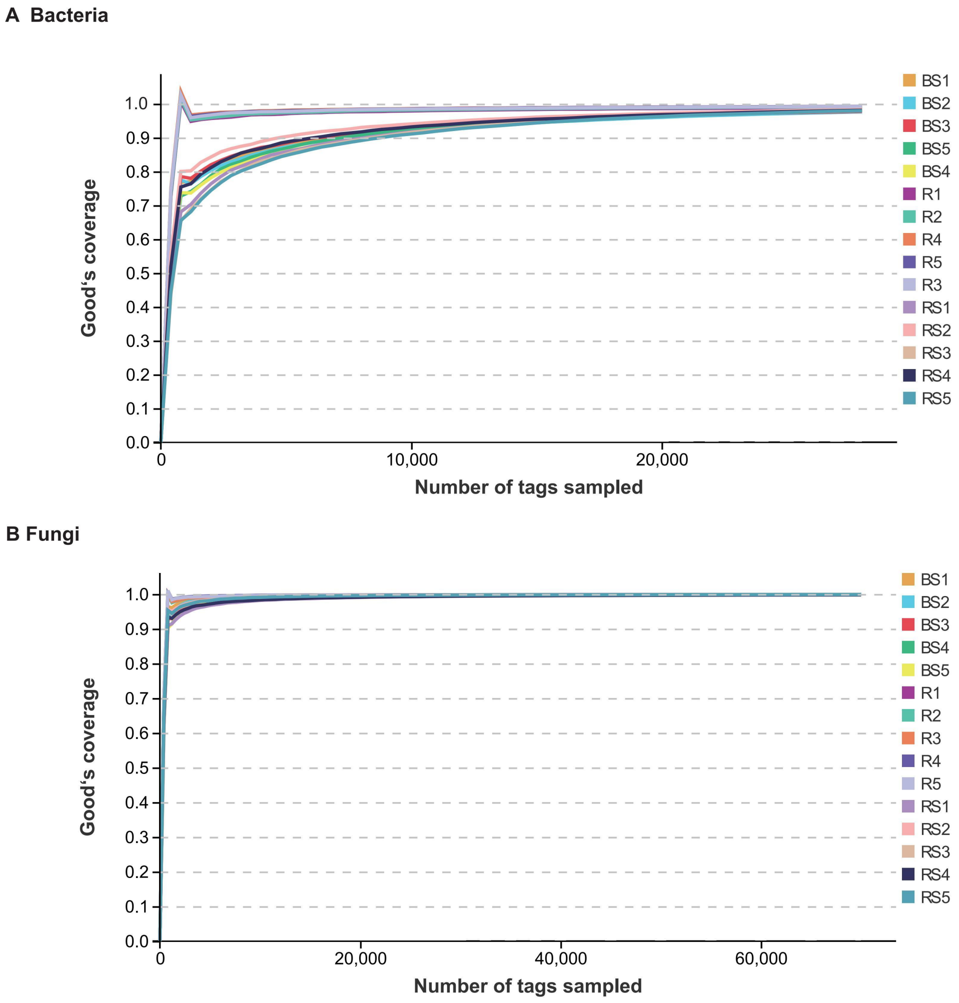

Figure S1. Rarefaction curves for the sequencing data of *Phellodendron amurense* roots (R), rhizosphere soil (RS) and bulk soil (BS). (**A**) Bacteria. (**B**) Fungi.

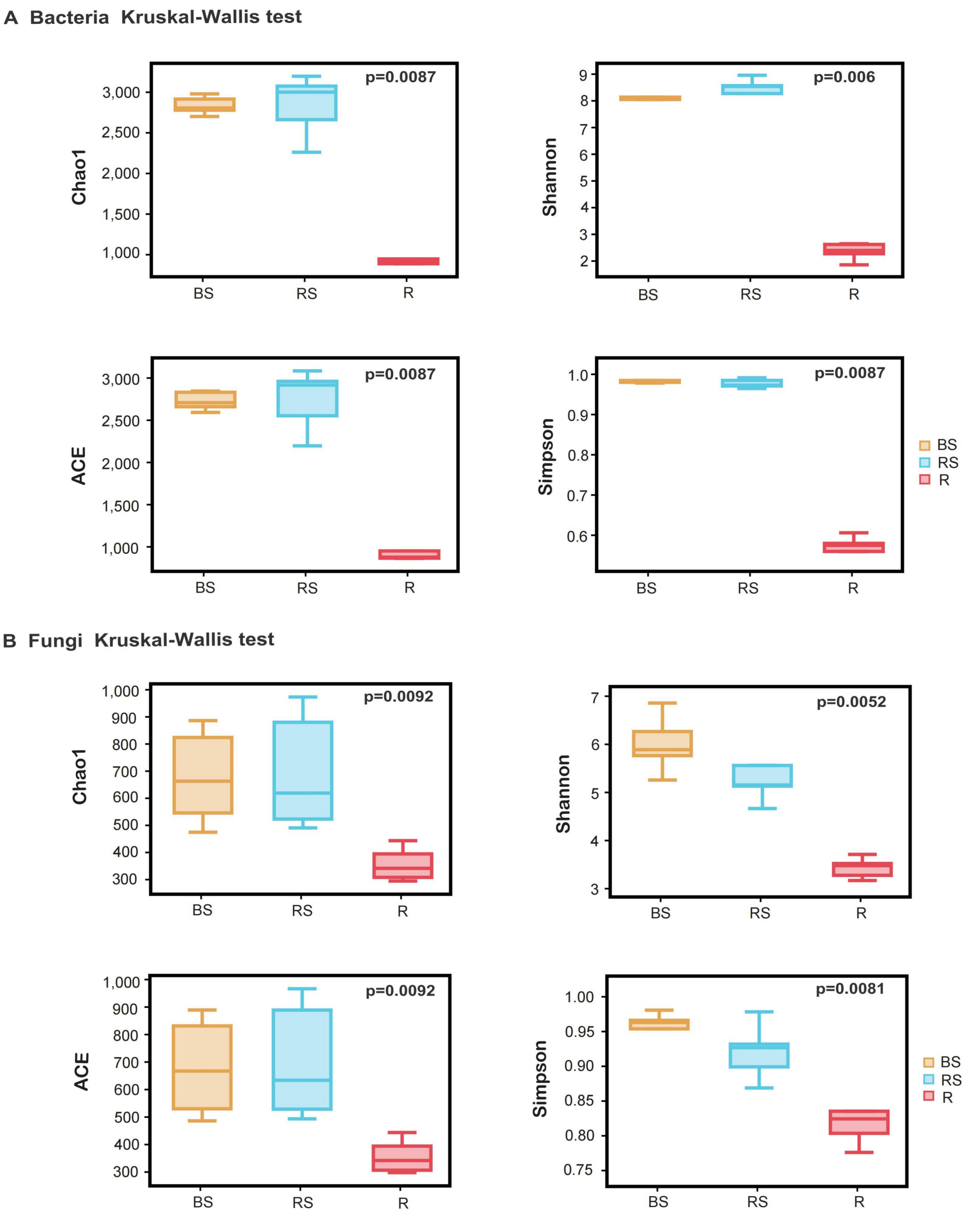

Figure S2. Alpha diversity of microbial communities associated with *Phellodendron amurense* (Kruskal-Wallis test). (**A**) Bacteria. (**B**) Fungi. BS, RS and R represent bulk soil, rhizosphere soil and roots, respectively.

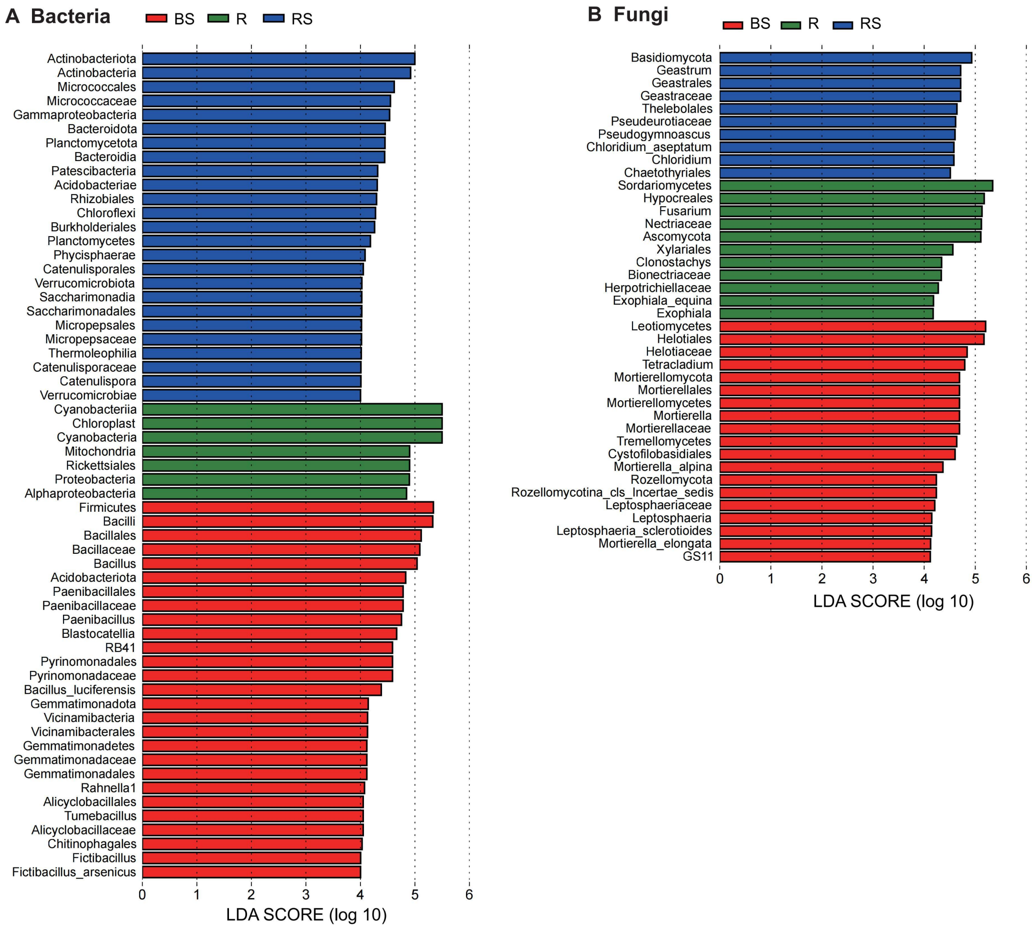

Figure S3. Linear discriminant analysis (LDA) aross roots (R), rhizosphere soil (RS) and bulk soil (BS) samples of *Phellodendron amurense*. All linear discriminant analysis (LDA) scores > 4. (**A**) Bacteria. (**B**) Fungi.
